# A phase II study of first-line afatinib for patients aged ≥75 years with *EGFR* mutation-positive advanced non-small cell lung cancer: North East Japan Study Group trial NEJ027

**DOI:** 10.1186/s12885-021-07861-1

**Published:** 2021-03-01

**Authors:** Yuji Minegishi, Ou Yamaguchi, Shunichi Sugawara, Shoichi Kuyama, Satoshi Watanabe, Kazuhiro Usui, Masahide Mori, Osamu Hataji, Toshihiro Nukiwa, Satoshi Morita, Kunihiko Kobayashi, Akihiko Gemma

**Affiliations:** 1 Department of Pulmonary Medicine and Oncology, Graduate School of Medicine, Nippon Medical School, 113-8603 1-1-5 Sendagi Bunkyo-Ku, Tokyo, Japan; 2 Department of Respiratory Medicine, Comprehensive Cancer Center, International Medical Center, Saitama Medical University, 1397 Yamane, Hidaka City, Saitama, 350-1298 Japan; 3 Department of Pulmonary Medicine, Sendai Kousei Hospital, 4-15 Hirose-machi, Aoba-ku, Sendai, 980-0873 Japan; 4 Department of Respiratory Medicine, National Hospital Organization, Iwakuni Clinical Center, 1-1-1 Atago Cyo, Iwakuni City, Yamaguchi Prefecture 740-8510 Japan; 5 Department of Respiratory Medicine and Infectious Diseases, Niigata University Graduate School of Medical and Dental Sciences, 1-757 Asahimachidori, Chuouku, Niigata, 951-8510 Japan; 6 Division of Respirology, NTT Medical Center Tokyo, 5-9-22 Higashigotanda Shinagawa, Tokyo, Japan; 7 Department of Thoracic Oncology, National Hospital Organization Osaka Toneyama Medical Center, 5-1-1 Toneyama, Toyonaka City, Osaka, 560-8552 Japan; 8 Respiratory Center, Matsusaka Municipal Hospital, 1550 Tonomachi, Matsusaka City, Mie 515-8544 Japan; 9 Tohoku University, 2-1 Seiryo-machi, Aoba-ku, Sendai, Miyagi 980-8575 Japan; 10 Department of Biomedical Statistics and Bioinformatics, Kyoto University Graduate School of Medicine, 54 Kawahara-cho, Shogoin, Sakyo-ku, Kyoto, 606-8507 Japan

**Keywords:** Non-small cell lung cancer, Elderly patients, Japan, Afatinib, *EGFR* mutation, Efficacy, Safety, Dose adjustment

## Abstract

**Background:**

Lung cancer is most common among older individuals. However, polypharmacy and comorbidities, which are also more common in older individuals, can limit treatment options. Previous studies suggest that afatinib can be used safely and effectively in elderly patients. This study investigated the anti-tumour activity and safety profile of first-line afatinib in previously-untreated elderly Japanese patients with *EGFR* mutation-positive non-small cell lung cancer (NSCLC).

**Methods:**

This was a single-arm, open-label, phase II study, performed in multiple centres in Japan. Previously untreated patients, aged ≥75 years, with *EGFR* mutation-positive (Del19 or L858R) advanced NSCLC were treated with afatinib 40 mg until disease progression or unacceptable toxicity. Adverse events (AEs) were managed with protocol-defined dose adjustments. The primary endpoint was objective response rate (ORR) by central review.

**Results:**

In total, 38 patients received at least one dose of afatinib, and 37 were evaluable for response. Median age was 77.5 years (range 75–91), all patients had an Eastern Cooperative Oncology Group performance status of 0 or 1, and 60.5% had Del19-positive disease. Median follow-up was 838 days. ORR was 75.7% (2 complete responses and 26 partial responses). Median progression-free survival was 14.2 months (95% confidence interval [CI], 9.5–19.0). Median overall survival (OS) was 35.2 months (95% CI, 35.2–not reached); the 2-year OS rate was 78.3%. The most common grade 3/4 treatment-related AEs (TRAEs) were diarrhoea (28.9%), paronychia (23.7%), and rash/acne (15.8%). Dose reductions due to TRAEs were reported in 78.9% of patients, and eight (21.1%) patients discontinued afatinib due to TRAEs. No treatment-related deaths were reported.

**Conclusion:**

Although dose adjustments were relatively common in this small group of Japanese patients aged ≥75 years with *EGFR* mutation-positive NSCLC, discontinuation occurred much less frequently, and most patients were able to stay on treatment for well over a year. Further, afatinib was associated with high response rates and prolonged PFS and OS.

**Trial registration:**

The trial is registered with Japan Registry of Clinical Trials (JRCT) as trial number 031180136 (date of initial registration: 19 February 2019), and the University Hospital Network (UMIN) as trial number 000017877 (date of initial registration: 11 June 2015).

**Supplementary Information:**

The online version contains supplementary material available at 10.1186/s12885-021-07861-1.

## Background

Lung cancer is the leading cause of cancer-related death worldwide, and among men in Japan [1]. Lung cancer is most frequently diagnosed between the ages of 65 and 74 years [2]. With the population of older patients diagnosed with non-small cell lung cancer (NSCLC) increasing, the choice of first-line treatment for older patients is an important decision, which may be complicated by age-related factors such as comorbidities and polypharmacy [3]. In patients without a treatable oncogene driver, current Pan-Asian clinical practice guidelines recommend carboplatin-based doublet chemotherapy for eligible patients aged ≥70 years with Eastern Cooperative Oncology Group performance status (ECOG PS) of 0–2 and adequate organ function, while single-agent chemotherapy remains the standard of care for patients not eligible for doublet chemotherapy [1]. In addition, the guidelines recommend considering immunotherapy for elderly patients who are not candidates for targeted therapy.

Epidermal growth factor receptor (EGFR) activation through tumour *EGFR* gene mutations drives malignancy in a proportion of patients with NSCLC. Activating *EGFR* mutations are found in up to 50% of NSCLC tumours from Asian populations, including in 30–40% of Japanese patients [4, 5]. Patients whose tumours harbour *EGFR* mutations may be sensitive to EGFR tyrosine kinase inhibitors (TKIs), which are recommended treatment options in this setting [1, 6, 7]. Asian treatment guidelines recommend EGFR-TKI monotherapy as first-line treatment for the general population of patients with *EGFR* mutation-positive NSCLC [1, 8], and there is evidence that EGFR-TKIs may be effective for some elderly patients with *EGFR* mutation-positive NSCLC [9].

Afatinib is a second-generation, irreversible ErbB-family blocker [10] that is approved in many countries, including Japan [11], for the first-line treatment of patients with *EGFR* mutation-positive NSCLC. The efficacy of afatinib in treating patients with *EGFR* mutation-positive NSCLC was established in the global, phase III LUX-Lung 3 study [12], the phase III LUX-Lung 6 study in Asian patients [13], and the global, phase IIb LUX-Lung 7 study [14, 15]. Afatinib was generally well tolerated in these studies; treatment-related adverse events (TRAEs) were mainly EGFR-TKI class-related toxicities, and were managed with tolerability-guided dose reductions. Few treatment discontinuations were reported [12, 13]. Among Japanese patients in LUX-Lung 3, prolonged progression-free survival (PFS) and improved overall survival (OS) in patients with *EGFR* Del19-positive tumours was confirmed for afatinib versus platinum-based chemotherapy [16]. The safety profile of afatinib in Japanese patients was as to be expected from EGFR-TKI exposure, but a higher rate of afatinib dose reductions was observed compared with the overall LUX-Lung 3 population (76% vs 52%) [12, 16]. In a subgroup analysis of the LUX-Lung 3, 6, and 7 studies, afatinib was effective and tolerable in patients with *EGFR* mutation-positive NSCLC, independent of age at diagnosis [3].

In addition to evidence from the LUX-Lung studies, a phase I trial including treatment at the approved dose of 40 mg, and a phase II trial employing afatinib at 30 mg support the feasibility of first-line afatinib as a treatment option in elderly Japanese patients with *EGFR* mutation-positive NSCLC [17, 18]. The aim of the current study was to further investigate the antitumour activity and safety profile of daily afatinib (40 mg) in previously untreated Japanese patients aged ≥75 years with *EGFR* mutation-positive NSCLC.

## Methods

### Study design

NEJ027 was a single-arm, multicentre, open-label, phase II study of first-line afatinib in patients aged ≥75 years, with *EGFR* mutation-positive NSCLC. The primary endpoint was objective response rate (ORR). Secondary endpoints were PFS, PFS in patients with *EGFR* Del19- versus L858R-positive disease, time to treatment failure (TTF), OS, disease control rate (DCR), 1- and 2-year survival rates, and frequency of adverse events (AEs).

The study was approved by the Institutional Review Boards of all participating institutions and was performed in accordance with the Declaration of Helsinki, the International Conference on Harmonisation of Technical Requirements for Pharmaceuticals for Human Use, Good Clinical Practice, and local laws. All patients provided written informed consent.

The trial is registered with the Japan Registry of Clinical Trials (JRCT) as trial number 031180136, and the University Hospital Network (UMIN) as trial number 000017877.

### Patients and treatment

Patients were aged ≥75 years, with histologically or cytologically confirmed stage III/IV disease (according to the General Rule for Clinical and Pathological Record of Lung Cancer, 7th edition) [19] or recurrent NSCLC, ≥1 measurable lesion (per Response Evaluation Criteria in Solid Tumours [RECIST] version 1.1) [20], and *EGFR* Del19- or L858R-positive disease (see Additional Methods). An ECOG PS of 0–1, and a life expectancy of > 3 months were required. Previous treatment with chemotherapy or EGFR-targeting therapy was not allowed, and patients with active lung disease, symptomatic brain metastases and hypersensitivity to the study drug were excluded.

Patients received afatinib at a starting dose of 40 mg/day until disease progression or unacceptable toxicity. Continuation of afatinib treatment after disease progression was permitted. Appropriate prevention and management of designated AEs (diarrhoea and skin disorders) was provided through treatment interruptions and dose reductions. Dose adjustments were implemented for Common Terminology Criteria for Adverse Events (CTCAE) grade ≥ 3 AEs or prolonged selected grade 2 AEs (including diarrhoea, stomatitis, rash, nausea, vomiting) despite best supportive care. During treatment interruptions, afatinib was suspended until AE severity recovered to grade ≤ 1 or baseline severity. If recovery was achieved within 14 days, afatinib was resumed at a lower dose by 10-mg decrements to a minimum of 20 mg/day; otherwise, dosing was permanently discontinued. If 20 mg/day was intolerable, afatinib was permanently discontinued. The duration of treatment interruption could be longer than 14 days provided the patient had sufficiently recovered from the AE within 14 days.

### Outcomes and assessments

Radiographic evaluation was performed every 8 weeks, and then every 12 weeks after 1 year. Response was assessed by extramural central review, including by the executive secretariat, according to RECIST version 1.1. Tumour response was defined as the proportion of patients with a complete response (CR) or partial response (PR). Tumour responses were confirmed by reassessment after 4 weeks.

PFS was defined as the time from registration until disease progression with first-line treatment, or death. OS was defined as time from registration until death. Best tumour response was defined without confirmation. Objective response was defined as CR or PR. Disease control was defined as CR, PR, or stable disease. Time to treatment failure (TTF) was defined as the time from registration until discontinuation of afatinib treatment, regardless of reasons such as disease progression, adverse event (AE), and death.

AEs were coded using the Medical Dictionary for Regulatory Activities (MedDRA) Japanese version 13.1 and graded using the CTCAE version 4.03 [21]. AEs were monitored throughout the study period, and worst grade was reported.

*EGFR* mutation analysis is described in the Additional Methods.

### Statistical considerations

Simon’s 2-stage minimax design was used to determine sample size. A minimum sample size of 35 patients was required based on the assumption that an expected ORR of > 70% would be clinically acceptable efficacy, and < 45% would be unacceptable (Additional Methods). These figures were determined based on studies available at the time the study was planned. Response rate in phase III trials comparing afatinib with conventional chemotherapy were 56–67% [12, 13], and 54–74% in elderly patients treated with first-generation EGFR-TKIs [22–24]. Thus, the expected response rate was determined to be 70%. In studies of patients aged ≥70 years receiving conventional cytotoxic chemotherapy, response rate was 23–54% [25–28]. It was therefore determined that a response rate of 45% would be the lower threshold response rate.

The safety analysis set (SAS) included all enrolled patients who received at least one dose of afatinib. The full analysis set (FAS) comprised all patients in the SAS, excluding those who had not been evaluated after starting treatment, and those who did not have an adequately evaluated lesion. Tumour response was evaluated in the FAS. Time-to-event data are descriptive and were estimated using Kaplan–Meier methodology (Additional Methods). The last date of confirmation of survival was 18 October 2019.

The collection, management, and statistical analysis of patient data was outsourced to an independent organisation without involvement of the researchers or sponsor.

### Role of the funding source

The study was funded by Nippon Boehringer Ingelheim Co. Ltd. The sponsor had no involvement in the study design, collection, analysis or interpretation of data, or in the decision to submit the article for publication.

## Results

### Patients

Between 28 January 2016, and 14 September 2017, 38 patients were enrolled and treated in the SAS. One patient was excluded with no appropriate measurable lesion; thus, the FAS comprised 37 patients (Additional Fig. 1). Patient follow-up was completed on 18 October 2019.

In the SAS population, the median age was 77.5 years (range 75–91), 15 (39.5%) patients were male, and all patients had an ECOG PS of 0–1 (Table 1). Most comorbidities were mild, with the most frequent baseline comorbidities being hypertension in 19 (50%) patients and hyperlipidaemia in 15 (39%) patients. Other complications included diabetes mellitus, thyroid disease, and chronic obstructive pulmonary disease, in two patients each. More than one-third of patients (34.2%) had brain metastases. *EGFR* Del19 was more common than L858R mutation. There were no marked differences in baseline characteristics between the SAS and FAS populations (Table 1).

**Table 1:** Patient and disease characteristics at baseline in the safety analysis set and full analysis set

Characteristic	Safety analysis set ***N =*** 38	Full analysis set ***N*** = 37
Sex		
Male	15 (39.5)	15 (40.5)
Female	23 (60.5)	22 (59.5)
Age, years		
Median (range)	77.5 (75–91)	77.0 (75–91)
Weight, kg		
Median (range)	50.2 (30.8–72.2)	50.5 (30.8–72.2)
BMI, kg/m^2^		
Median (range)	21.0 (16.5–26.0)	21.2 (16.5–26.0)
Smoking status		
Never	26 (68.4)	25 (67.6)
Former/current	12 (31.6)	12 (32.4)
ECOG PS		
0	21 (55.3)	20 (54.1)
1	17 (44.7)	17 (45.9)
Histological classification		
Adenocarcinoma	38 (100)	37 (100)
Clinical stage at study entry		
IIIB	1 (2.6)	1 (2.7)
IV	28 (73.7)	28 (75.7)
Postoperative recurrence	9 (23.7)	8 (21.6)
Comorbidities		
Yes	32 (84.2)	31 (83.8)
No	6 (15.8)	6 (16.2)
Metastases at study entry		
No distant metastases	4 (10.5)	4 (10.8)
Lung	10 (26.3)	10 (27.0)
Bone	13 (34.2)	13 (35.1)
Post palliative RT	1 (2.6)	1 (2.7)
Brain	13 (34.2)	13 (35.1)
Post palliative SRS	3 (7.9)	3 (8.1)
Liver	4 (10.5)	4 (10.8)
Pleural	11 (28.9)	11 (29.7)
*EGFR* mutation categories		
Del19	23 (60.5)	22 (59.5)
L858R	15 (39.5)	15 (40.5)

Data are *n* (%) unless otherwise stated

Abbreviations: *BMI* body mass index, *ECOG PS* Eastern Cooperative Oncology Group performance status, *EGFR* epidermal growth factor receptor, *RT* radiotherapy, *SRS* stereotactic radiosurgery

### Antitumour activity

Tumour response is summarised in Table 2 (Additional Fig. 2 details individual patient responses for all patients in the FAS except one patient with progressive disease due to a new brain metastasis for whom the target lesion was not evaluated). The ORR was 75.7% (95% confidence interval [CI], 58.8–88.2) and was not markedly different in patients with *EGFR* Del19- versus L858R-positive tumours (72.7% vs 80.0%). The DCR was also similar between these subgroups (86.4% vs 93.3%).

**Table 2:** Tumour response

Parameter	Full analysis set ***N =*** 37	***EGFR*** mutation	
		Del19 ***n =*** 22	L858R ***n =*** 15
Best response, *n* (%)			
CR	2 (5.4)	2 (9.1)	0
PR	26 (70.3)	14 (63.6)	12 (80.0)
SD	5 (13.5)	3 (13.6)	2 (13.3)
PD	2 (5.4)	1 (4.5)	1 (6.7)
NE	2 (5.4)	2 (9.1)	0
ORR, *n* (%) (95% CI)	28 (75.7) (58.8–88.2)	16 (72.7) (49.8–89.3)	12 (80.0) (51.9–95.7)
DCR, *n* (%) (95% CI)	33 (89.2) (74.6–97.0)	19 (86.4) (65.1–97.1)	14 (93.3) (68.1–99.8)

Abbreviations: *CI* confidence interval, *CR* complete response, *DCR* disease control rate, *EGFR* epidermal growth factor receptor, *NE* not evaluable, *ORR* objective response rate, *PD* progressive disease, *PR* partial response, *SD* stable disease

**Fig. 1:**
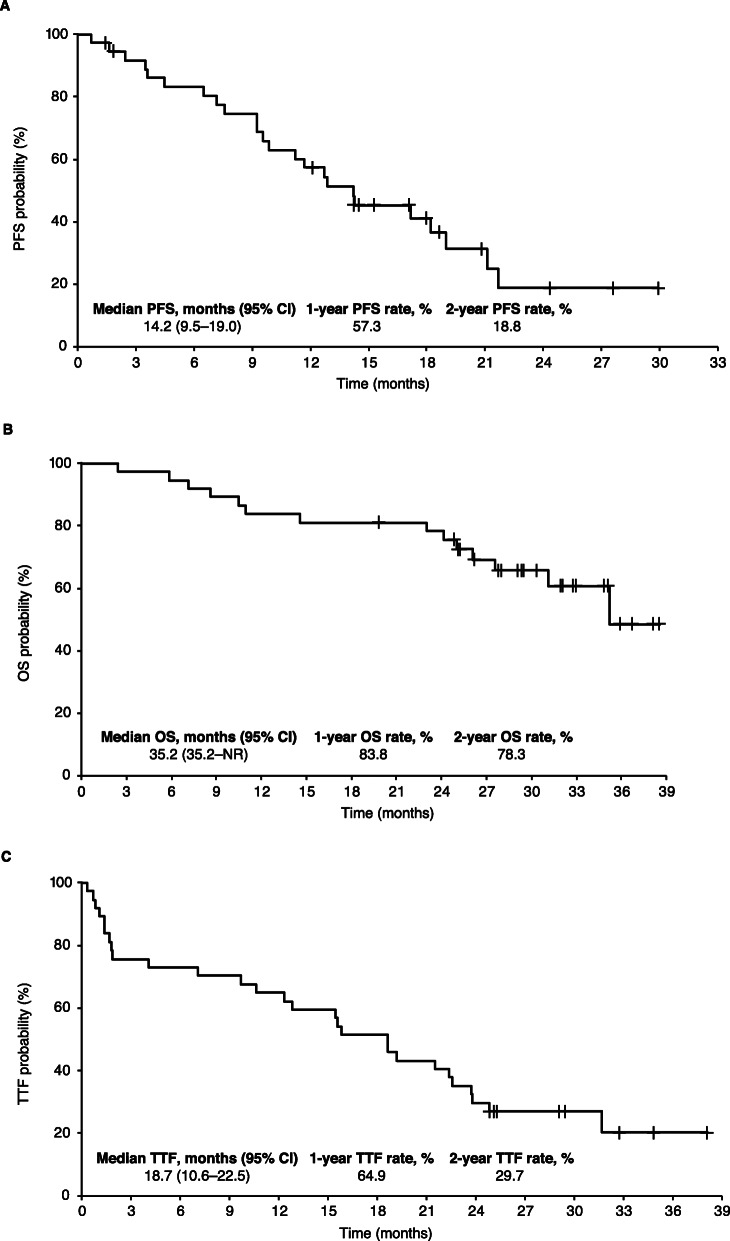
Kaplan–Meier survival analysis in the full analysis set (*n =* 37). **a** Progression-free survival (PFS)^a^. **b** Overall survival (OS). **c** Time to treatment failure (TTF). Abbreviations: *CI* confidence interval, *NR* not reached, *QOD* every other day. ^a^PFS was censored for four patients receiving afatinib < 20 mg/day. These patients were treated as censored when they fell below the 20 mg/day minimum dose specified in the protocol

The median follow-up period was 838 days (range 73–1156). Median PFS was 14.2 months (95% CI, 9.5–19.0). Median OS was 35.2 months (95% CI, 35.2–not reached), and 1- and 2-year OS rates were 83.8 and 78.3%, respectively. Median TTF was 18.7 months (95% CI, 10.6–22.5; Fig. 1).

In patients with *EGFR* Del19- or L858R-positive tumours, median PFS was 18.2 months and 12.9 months, respectively (Additional Fig. 3). Median OS was not reached in patients with Del19-positive tumours and was 35.2 months in those with L858R-positive disease; 2-year OS rates were 77.0 and 80.0%, respectively. The median TTF was 18.6 months in patients with Del19-positive tumours and 19.2 months in those with L858R-positive disease.

No significant differences in median PFS (21.4 vs 12.5 months; HR, 1.9; *p* = 0.14) or OS (not reached [NR] vs 34.8 months; HR, 0.9; *p* = 0.9) were observed between females (*n* = 15) versus males (*n* = 22). Similarly, median PFS (14.1 vs 11.0 months; HR, 1.3; *p* = 0.6) and OS (NR vs 34.8 months; HR, 0.9; *p* = 0.9) were also similar in non-smokers (*n* = 25) versus current/former smokers (*n* = 12) and patients with ECOG PS of 0 (*n* = 20) versus 1 (*n* = 17) (median PFS: 14.1 vs 11.0 months; HR, 1.4; *p* = 0.4; median OS: NR vs 34.8 months; HR, 2.2; *p* = 0.1). Median PFS (16.9 vs 3.5 months; HR, 0.20; *p* = 0.001) and OS (NR vs 10.3 months; HR, 0.09; *p* < 0.001) were significantly longer in patients who received a dose modification (*n* = 29) compared to those who did not (*n* = 8).

Patients with no comorbidities (*n* = 6) had numerically longer median PFS (16.9 vs 12.7 months; HR 1.8; *p* = 0.3) and OS (NR vs 34.8; HR, 1.3; *p* = 0.8) than did those with at least one comorbidity (*n* = 31), however the difference was not statistically significant.

### Treatment exposure and safety

**Fig. 2:**
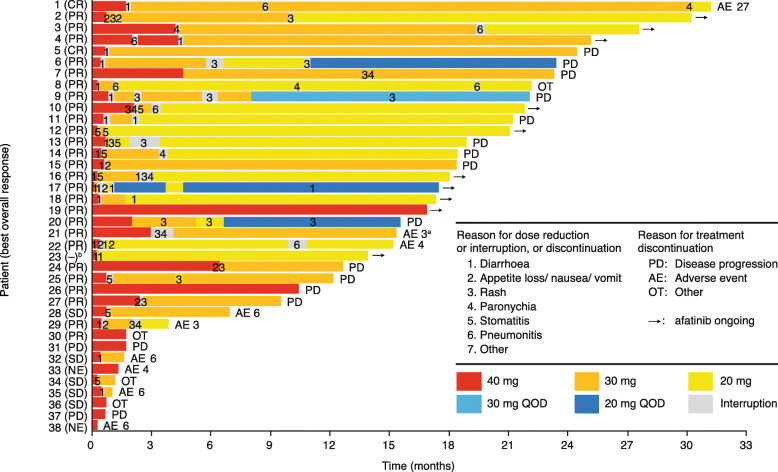
Treatment duration and afatinib dose in the safety analysis set (*n =* 38). Abbreviations: *AE* adverse event, *CR* complete response, *NE* not evaluable; *OT* other, *PD* progressive disease, *PR* partial response; *QOD* every other day, *SD* stable disease. ^a^For Patient 21, protocol treatment was discontinued due to disease progression; Thereafter, afatinib treatment beyond PD was discontinued due to skin disorders. ^b^Patient 23 had no appropriate measurable lesion and was not included in the full analysis set

In the SAS, median duration of treatment was 494 days (range 8–950). Thirty (78.9%) patients required at least one dose reduction of afatinib (Table 3). Treatment duration and afatinib dosage in individual patients is summarised in Fig. 2. Twenty-eight patients discontinued afatinib treatment, mainly due to disease progression (15 patients). At data cut-off, nine patients remained on treatment without disease progression, including the one patient excluded from the FAS, and one patient treated at < 20 mg (Additional Fig. 1).

**Table 3:** Afatinib exposure and treatment adjustment due to treatment-related adverse events

Category	Safety analysis set ***N =*** 38
Afatinib exposure	
Median treatment duration^a^, days (range)	494 (8–950)
Median treatment days^b^, n (range)	492 (8–932)
Mean afatinib dose^c^, mg	29.7 (10.6–40.0)
Median relative dose intensity^d^, % (range)	74.2 (26.4–100)
Treatment adjustments, *n* (%)	
Initial treatment dose 40 mg	38 (100)
Dose reduction	30 (78.9)
Final treatment dose	
40 mg	8 (21.1)
30 mg	12 (31.6)
20 mg	14 (36.8)
30 mg QOD	1 (2.6)
20 mg QOD	3 (7.9)
Treatment interruption	28 (73.7)
Treatment discontinuation	8 (21.1)

Abbreviation: *QOD* every other day, *TRAE* treatment-related adverse events

Data are *n *(%) unless otherwise stated. In addition to afatinib exposure, data for dose reductions, treatment interruption and discontinuation due to TRAEs are shown

^a^From start of treatment to discontinuation or censoring, including days of treatment interruption

^b^Not including treatment interruption days

^c^Total afatinib dose/treatment duration

^d^(mean afatinib dose/40) × 100

TRAEs are summarised in Table 4. Grade 3/4 TRAEs were reported in 28 (73.7%) patients; the most common were diarrhoea (11 [28.9%]), paronychia (nine [23.7%]), and rash/acne (six [15.8%]). Thirty (78.9%) patients had TRAEs leading to afatinib dose reductions, and 28 (73.7%) had TRAEs leading to afatinib treatment interruptions; the most common reasons for dose reductions or interruptions were diarrhoea (26 events) and rash/acne (20 events; Fig. 2). Eight (21.1%) patients discontinued treatment due to TRAEs; four had pneumonitis, two had paronychia, one had rash, and one had appetite loss and oedema. There were 14 recorded deaths, none of which was treatment related. Thirteen patients died due to lung cancer progression and one from debility due to dementia progression.

**Table 4:** Treatment-related adverse events

Adverse event	Any grades	Grade 1 or 2	Grade 3 or 4
Any	38 (100)	10 (26.3)	28 (73.7)
Diarrhoea	36 (94.7)	25 (65.8)	11 (28.9)
Rash/acne^a^	30 (78.9)	24 (63.2)	6 (15.8)
Paronychia	26 (68.4)	17 (44.7)	9 (23.7)
Stomatitis	26 (68.4)	21 (55.3)	5 (13.2)
Appetite loss	13 (34.2)	8 (21.1)	5 (13.2)
Vomiting	6 (15.8)	5 (13.2)	1 (2.6)
Pneumonitis	5 (13.2)	3 (7.9)	2 (5.3)^b^
Fatigue	5 (13.2)	5 (13.2)	0
Nausea	4 (10.5)	4 (10.5)	0
Oedema	4 (10.5)	4 (10.5)	0
Infection	4 (10.5)	3 (7.9)	1 (2.6)
ALT/AST increased	9 (23.7)	8 (21.1)	1 (2.6)
Creatinine increased	8 (21.1)	8 (21.1)	0
Anaemia	8 (21.1)	7 (18.4)	1 (2.6)
Hypoalbuminemia	8 (21.1)	8 (21.1)	0
Thrombocytopenia	6 (15.8)	6 (15.8)	0
Hypokalaemia	6 (15.8)	5 (13.2)	1 (2.6)
Leukocytopenia	4 (10.5)	4 (10.5)	0

Abbreviations: *ALT* alanine aminotransferase, *AST* aspartate aminotransferase, *TRAE* treatment-related adverse event

Data are *n* (%) TRAEs in > 10% of patients in the safety analysis set (*n =* 38) listed by Medical Dictionary for Regulatory Activities (MedDRA) preferred terms and grade by Common Terminology Criteria for Adverse Events (CTCAE), version 4.0

^a^Includes papulopustular rash, rash pustular, and rash acneiform

^b^Includes one patient with grade 4 pneumonitis

### Treatment after disease progression

Twelve patients with disease progression continued afatinib beyond progression for > 30 days, and one was still receiving afatinib at data cut-off. Of the 28 patients who discontinued afatinib, eight did not receive any further treatment, while 20 received second-line treatments (osimertinib *n* = 5, other EGFR-TKIs *n* = 7, cytotoxic agents alone *n =* 5, platinum-doublet chemotherapy *n* = 2, pembrolizumab *n* = 1). In total, eight patients received osimertinib during the observation period (three in third or later lines).

## Discussion

In this study, we examined elderly patients aged ≥75 years (median [range] 77.5 [75–91] years); this has been identified previously as a relevant cut-off when considering the age at which more effective and tolerable therapies compared with chemotherapy are needed [3, 29]. Baseline patient and disease characteristics were largely similar to those reported in two previous investigations of afatinib in Japanese patients aged ≥75 years (median [range] 79 [75–87] years) [17] and > 70 years (median [range] 77 [70–85] years) [18], and in a subgroup analysis of older patients in the LUX-Lung 7 study (median [range] 79 [75–86] years) [3]. However, the frequency of patients with Del19-positive disease (61%) was slightly higher than reported in elderly patients in the other two Japanese studies (20 to 55%) [17, 18], and in the LUX-Lung 7 subanalysis (37%) [3]. Most patients (84%) in the current study had comorbidities, the most common being hypertension, and more than one third (34%) had baseline brain metastases.

The ORR was 75.7%, and the primary endpoint of the study was met. Clinical activity of afatinib with respect to response and other efficacy outcomes (PFS and OS) was encouraging, consistent with other studies in elderly Japanese patients treated with afatinib in the same setting [17, 18]. Efficacy outcomes were also similar in patients with *EGFR* Del19- and L858R-positive tumours, as reported previously [18].

The safety profile of afatinib was as expected from EGFR-TKI treatment in elderly Japanese patients [16, 18, 30], and was manageable with dose reductions. Previous data suggest that Japanese patients are more likely to develop pneumonitis than patients of other nationalities when treated with EGFR-TKIs [31]. In the current study, five (13.2%) patients had treatment-related pneumonitis (one grade 3 and one grade 4), four of whom discontinued treatment. Similarly, in a previous phase II study of elderly Japanese patients treated with afatinib, pneumonitis was reported in four (10%) patients, one of whom had grade 3 pneumonitis and two of whom died whilst on treatment, suggesting that elderly patients treated with an EGFR-TKI should be monitored for pneumonitis [18].

The incidence of grade 3/4 TRAEs was 73.7%; this is considerably higher than the rate seen with afatinib in LUX-Lung 7 (31%; median age 63 years) [14], suggesting that patients of older age more often experience severe adverse events than younger patients with largely similar ECOG PS (0/1: 54/46% [this study] vs 32/68% [LUX-Lung 7]). Although grade 3/4 TRAEs occurred more frequently in this older population, response and survival outcomes were numerically better in our study than in LUX-Lung 7 (median PFS 14.2 vs 11.0 months; median OS 35.2 vs 27.9 months; ORR 75.7% vs 70%) [14, 15]. In a non-interventional study involving more than 1600 Japanese patients (median age, 67 years; ECOG PS, 0/1 40/46%), grade 3/4 adverse drug reactions were reported in 36% of patients; however, ORR was much lower (40%; survival was not reported) [30].

The starting dose of afatinib in the current study was 40 mg; however, a previous phase II study in elderly Japanese patients reported acceptable tolerability and encouraging antitumour activity with a starting dose of 30 mg afatinib [18]. This raises discussion on whether the afatinib starting dose should be lower than 40 mg in older Japanese patients, and as low as 20 mg in some instances [18]. Our finding that patients who underwent dose reductions had longer PFS and OS than those who did not also suggests that a lower dose may be preferable. This latter finding, however, should be interpreted with caution. It is likely that survival was shorter in the higher-dose group because most of this group discontinued treatment early due to disease progression or adverse events. As a result of their shorter time on treatment, these patients were less likely to undergo a dose reduction. Given the high possibility of bias due to early discontinuation of afatinib, these data do not necessarily support the use of a reduced starting dose of afatinib.

While dose reductions due to AEs were more frequent in the current study compared with the previous phase II study (79% vs 48%), rates of treatment discontinuation were comparable (21% vs 20%). Post-hoc analyses of LUX-Lung 3, 6, and 7 showed that reductions from the afatinib 40 mg starting dose had no effect on therapeutic efficacy [32–34]. However, our findings of promising efficacy and manageable toxicity with afatinib 40 mg may argue against using a reduced starting dose in elderly Japanese patients with good performance status. Instead, starting at 40 mg and employing dose reductions may mitigate the effects of potential interpatient differences in drug pharmacokinetics/pharmacodynamics, while reducing the possibility of under treatment at lower afatinib doses. Further, using a standard dose of afatinib in elderly individuals with good performance status will simplify treatment planning and administration, and reduce the possibility of under-dosing. Use of the established tolerability-guided dose reduction protocol allows for rapid and flexible dose adjustments, helping patients remain on a higher dose for longer.

There is accumulating evidence that TKIs may be more effective in elderly than in younger patients with *EGFR* mutation-positive NSCLC [9, 33]. Moreover, given that kinase inhibitors usually show milder toxicity than cytotoxic chemotherapy, the current Japanese Lung Cancer Society guideline for stage IV NSCLC recommends (level 1C) the use of any EGFR-TKI for the first-line treatment of elderly patients with a driver oncogene [8]. In this regard, afatinib may be a suitable choice for elderly patients with *EGFR* mutation-positive NSCLC who are receiving multiple concomitant medications, due to its reported low potential for drug–drug interactions, and low exposure to hepatic metabolism and excretion [35].

The interpretation of this analysis should be treated with caution due to inherent limitations of this type of study, including its single-arm design, which precluded randomisation, and retrospective nature, which meant that patients were not blinded to treatment. Further, only small numbers of patients were investigated overall and in each *EGFR* mutation subgroup. This is particularly true for the subgroup analyses that investigated the impact of sex, smoking status, ECOG PS, dose modifications, and comorbidities on survival. Due to the small numbers of patients in some of these subgroups, the absolute values should be interpreted with caution. Perhaps most importantly, ECOG performance status was 0–1 in all patients, which resulted in a selection bias in favour of patients who may be likely to have a better outcome from treatment.

## Conclusions

Although dose adjustments were relatively common in this small group of Japanese patients aged ≥75 years with *EGFR* mutation-positive NSCLC, discontinuation occurred less frequently, and most patients were able to stay on treatment for well over a year. Further, afatinib was associated with high response rates and prolonged PFS and OS. These findings support the use of first-line afatinib at a starting dose of 40 mg in elderly *EGFR* mutation-positive patients with NSCLC.

## Supplementary Information

**Additional file 1 **: **Additional methods** (patient inclusion and exclusion criteria, *EGFR* mutation detection, statistical considerations), **Additional Fig. 1** (patient disposition), **Additional Fig. 2** (individual patient best overall response in the safety analysis set [*n* = 38]. Shown is a waterfall plot of maximum tumour reduction from baseline assessed according to RECIST version 1.1 criteria in 36 patients. Confirmation of response could not be obtained for two non-evaluable patients, one with no evaluable target lesion and one with confirmed progressive disease due to a new brain metastasis), **Additional Fig. 3** (Kaplan–Meier survival analysis by *EGFR* mutation type in the full analysis set [*n* = 37]. (A) Progression-free survival [PFS]. (B) Overall survival [OS]. (C) Time to treatment failure [TTF]).

## Data Availability

The datasets used and/or analysed during the current study are available on reasonable request from the corresponding author.

## Abbreviations

*EGFR*: Epidermal growth factor receptor
*TKI*: Tyrosine kinase inhibitors
*NSCLC*: Non-small cell lung cancer
*AE*: Adverse event
*ORR*: Objective response rate
*DCR*: Disease control rate
*PFS*: Progression-free survival
*OS*: Overall survival
*TTF*: Time to treatment failure
*TRAE*: Treatment-related AE
*del19*: *EGFR* exon 19 deletions
*ECOG*: Eastern Cooperative Oncology Group
*PS*: Performance status
*RECIST*: Response Evaluation Criteria in Solid Tumours
*CTCAE*: Common Terminology Criteria for Adverse Events
*CR*: Complete response
*PR*: Partial response
*NE*: Not evaluable
*PD*: Progressive disease
*SD*: Stable disease
*CI*: Confidence interval
*MedDRA*: Medical Dictionary for Regulatory Activities
*SAS*: Safety analysis set
*FAS*: Full analysis set
*BMI*: Body mass index
*RT*: Radiotherapy
*SRS*: Stereotactic radiosurgery
*QOD*: Every other day
*ALT*: Alanine aminotransferase
*AST*: Aspartate aminotransferase
*NR*: Not reached
