# Supplementary material for: A phase II study of first-line afatinib for patients aged ≥75 years with EGFR mutation-positive advanced non-small cell lung cancer: North East Japan Study Group trial NEJ027
Source: BMC Cancer. 2021 Mar 1;21:208. doi: 10.1186/s12885-021-07861-1 (PMC7919080; doi:10.1186/s12885-021-07861-1)
Supplement: Supplementary file 1 — Additional file 1 : Additional methods (patient inclusion and exclusion criteria, EGFR mutation detection, statistical considerations), Additional Fig. 1 (patient disposition), Additional Fig. 2 (individual patient best overall response in the safety analysis set [n = 38]. Shown is a waterfall plot of maximum tumour reduction from baseline assessed according to RECIST version 1.1 criteria in 36 patients. Confirmation of response could not be obtained for two non-evaluable patients, one with no evaluable target lesion and one with confirmed progressive disease due to a new brain metastasis), Additional Fig. 3 (Kaplan–Meier survival analysis by EGFR mutation type in the full analysis set [n = 37]. (A) Progression-free survival [PFS]. (B) Overall survival [OS]. (C) Time to treatment failure [TTF]). [file 12885_2021_7861_MOESM1_ESM.docx]

**Additional material**

**Additional methods**

**Patient inclusion and exclusion criteria**

- Patient inclusion criteria:
  - Histologically or cytological confirmed stage III/IV non-small cell lung cancer (NSCLC) (defined according to the 7^th^ edition of the General Rule for Clinical and Pathological Record of Lung Cancer) [1] or recurrent disease.
  - Patients with tumours harbouring an epidermal growth factor receptor (*EGFR*) Del19 or L858R mutation (excluding patients whose tumours harboured uncommon mutations such as T790M, in combination with *EGFR* Del19 or L858R mutations).
  - No prior chemotherapy and no prior treatment with EGFR-targeting small molecules or antibodies.
  - Criteria regarding pretreatments and washout periods before entry included: ≥12 months since the day of final administration of preoperative or postoperative adjuvant chemotherapy; ≥2 weeks since the last pleurodesis that did not use anticancer drugs; ≥2 weeks must have elapsed since the systemic administration of steroids for any period of time exceeding 4 weeks; and ≥4 weeks since the administration of other investigational drugs.
  - ≥1 measurable lesion per Response Evaluation Criteria in Solid Tumours (RECIST), version 1.1.
  - Aged ≥75 years.
  - Eastern Cooperative Oncology Group performance status 0 or 1
  - Adequate organ function, including:
    - Neutrophil count ≥1,500/mm^3^.
    - Haemoglobin ≥9.0 g/dL.
    - Platelet count ≥7.5 x 10^4^/mm^3^.
    - Total bilirubin ≤1.5 times the upper limit of the site’s standard values.
    - Aspartate transaminase/alanine transaminase ≤3.0 times the upper limit of the site’s standard values.
    - Serum creatinine ≤1.5 times the upper limit of the site’s standard values.
    - PaO_2_ (room air) ≥60 Torr, or SpO_2_ ≥93%.
  - Life expectancy > 3 months.
  - Written informed consent.
- Patient exclusion criteria:
  - Chemotherapy, biological therapy, or investigational agents for other carcinomas within 4 weeks prior to the start of study treatment.
  - Hormonal treatment within 2 weeks prior to start of study treatment.
  - Previous radiotherapy to the primary tumour or measurable lesion.
  - Major surgery within 4 weeks before starting study treatment, or scheduled for surgery during the projected course of the study.
  - Complications with concurrent cancers that have a disease-free period of <5 years.
  - Active lung disease such as interstitial pneumonia, active radiation pneumonitis, or drug-induced pneumonitis.
  - Symptomatic brain metastases (patients with asymptomatic brain metastasis were eligible to enrol in the study).
  - Any other concurrent illnesses or conditions and/or infections that could interfere with the conduct of the trial.
  - Women who are currently pregnant or breastfeeding.
  - Known hypersensitivity to afatinib or the excipients of any of the trial drugs.
  - Individuals who are deemed to be unsuitable for participation in this study by the principal investigator or sub-investigators, due to comorbidity of mental disorders such as dementia, or for any other reason (cognitive decline is a particularly important exclusion criterion).

***EGFR* mutation detection**

*EGFR* mutation analysis was performed on DNA extracted from paraffin-embedded tissue sections or cytological specimens. A definitive diagnosis of the presence or absence of activating *EGFR* mutations was made during the course of routine medical care using a test covered by the patient’s health insurance, including high sensitivity detection methods such as the peptide nucleic acid-locked nucleic acid polymerase chain reaction clamp method.

**Statistical considerations**

A sample size of 35 patients was required based on the assumption that an expected objective response rate (ORR) of >70% would be clinically acceptable efficacy, and <45% would be unacceptable. For an α error = 0.05, β error = 0.1 and one-sided tests, 33 patients were required. Allowing approximately 10% loss of patients in follow-up, a total of 35 patients were planned to be enrolled. Afatinib administered at <20 mg was considered to be a deviation from the protocol. Progression-free survival (PFS) was censored in patients treated with <20 mg afatinib.

**Reference**

1. The Japan Lung Cancer Society. General rule for clinical and pathological record of lung cancer, 7th edition. Kanehara & Co, Tokyo, Japan; 2010.

**Additional Fig. 1** Patient disposition

**
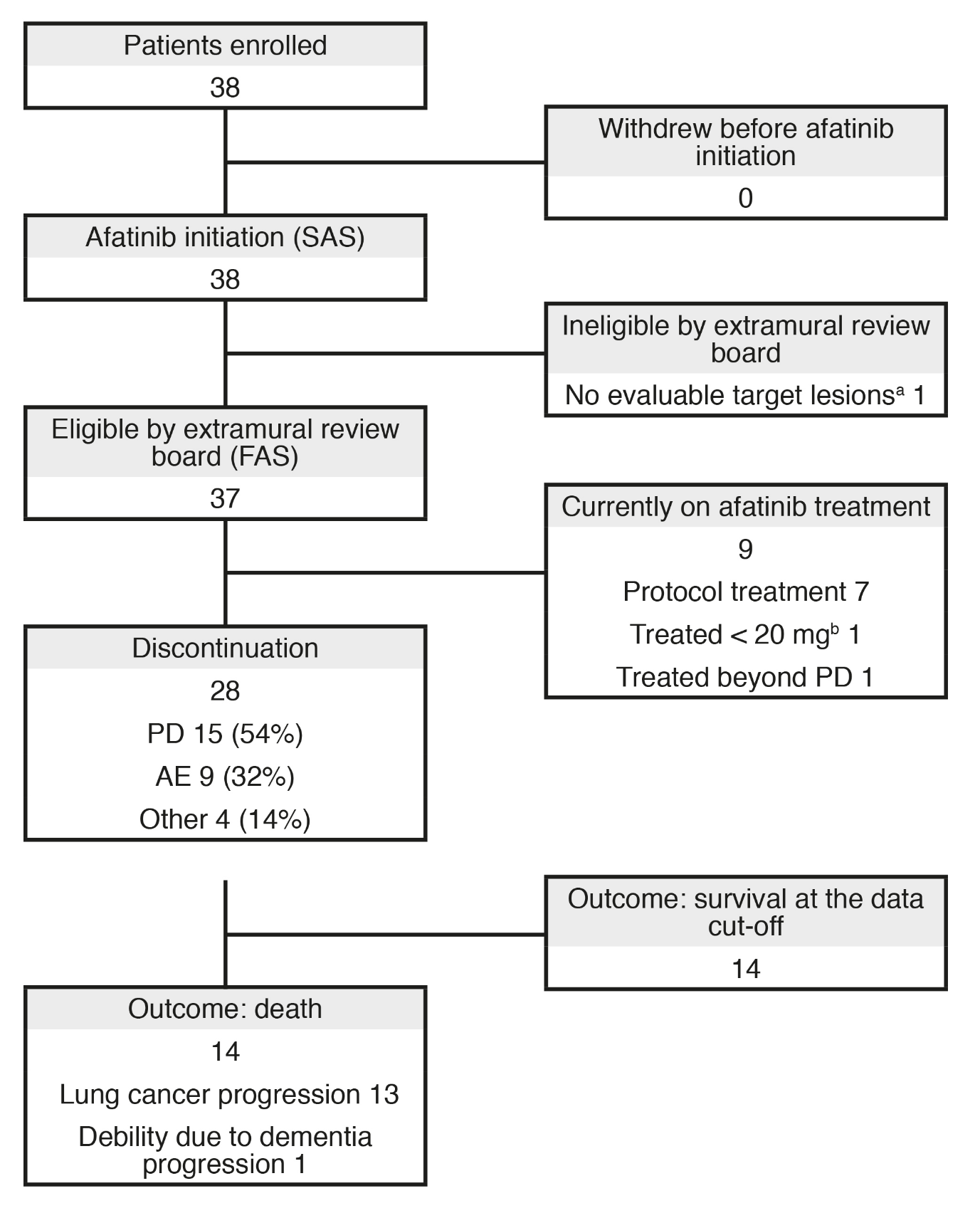
**

Abbreviations: *AE* adverse event, *FAS* full analysis set, *PD* progressive disease, *SAS* safety analysis set.

^a^Currently on PD (Figure 2, Patient 23); ^b^Protocol violation.

**Additional Fig. 2** Individual patient best overall response in the safety analysis**
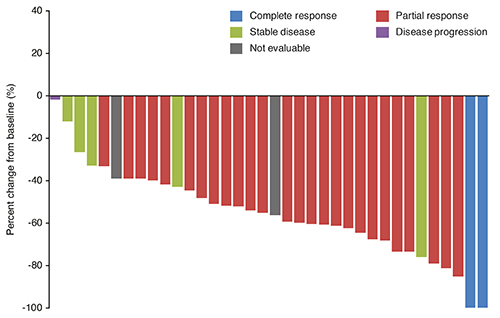
** set (n = 38). Shown is a waterfall plot of maximum tumour reduction from baseline assessed according RECIST v1.1 criteria in 36 patients. Confirmation of response could not be obtained for 2 non-evaluable patients, one with no evaluable target lesion and one with confirmed progressive disease due to a new brain metastasis

Abbreviation: *RECIST* Response Evaluation Criteria in Solid Tumours.

**Additional Fig. 3** Kaplan–Meier survival analysis by *EGFR* mutation type in the full analysis set (n = 37). (A) Progression-free survival (PFS). (B) Overall survival (OS). (C) Time to Treatment failure (TTF)**
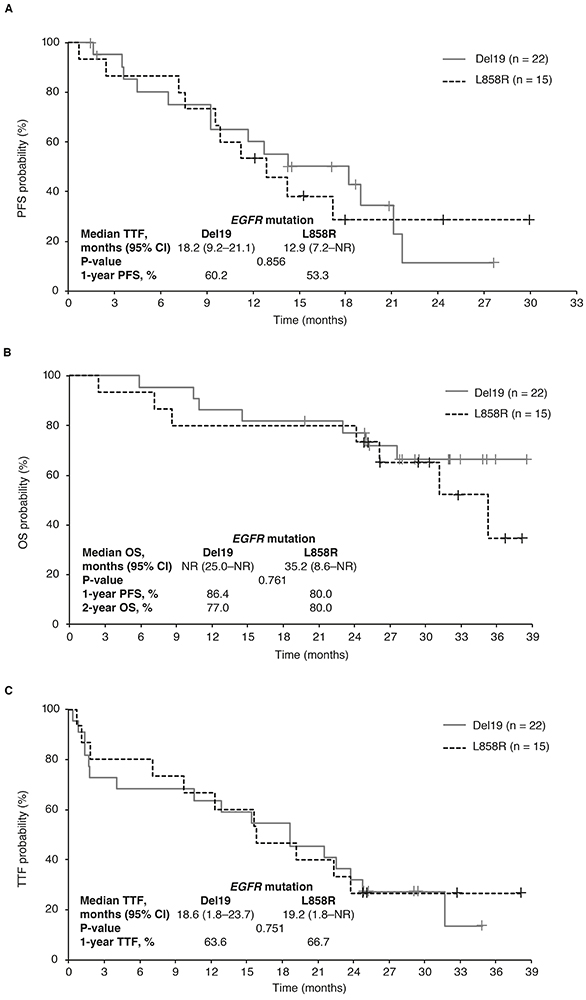
**

Abbreviations: *CI* confidence interval, *EGFR* epidermal growth factor, *NR* not reached.
